# Simultaneous Administration of Dexamethasone and Vitamin E Reversed Experimental Varicocele-induced Impact in testicular tissue in Rats; Correlation with Hsp70-2 Chaperone Expression

**DOI:** 10.1590/S1677-5538.IBJU.2013.0148

**Published:** 2015

**Authors:** Hajar Khosravanian, Mazdak Razi, Farah Farokhi, Narges Khosravanian

**Affiliations:** 1 Department of Biology, Faculty of Basic Science, Urmia University, Urmia, Iran; 2 Department of Comparative Histology & Embryology, Faculty of Veterinary Medicine, Urmia University, Urmia, Iran

**Keywords:** Varicocele, Hsp70-2 chaperone, Nitrosative stress, Oxidative stress, Inflammation, Dexamethasone, Vitamin E

## Abstract

**Purpose:**

This study aimed to investigate the protective effects of isolated and co-administration of vitamin E (VitE) and dexamethasone (DEX) on varicocele (VCL)-induced damages in testicular tissue.

**Materials and Methods:**

Wistar rats were divided into five groups (n=6), including; control-sham, non-treated VCL-induced, VitE-treated VCL-induced (VitE, 150 mg/kg, orally), DEX-administrated VCL-induced (DEX, 0.125 mg/kg, i.p.), VitE+DEX-received VCL-induced animals. The antioxidant status analyses, histopathological examinations, hormonal assay and tissue levels of alkaline phosphatase (ALP) were analyzed. The germinal epithelium RNA damage and Leydig cells steroidogenesis were analyzed. Moreover, the Hsp70-2 protein expression was examined based on immunohistochemical and western blot analyses. The sperm parameters, DNA integrity and chromatin condensation were investigated.

**Results:**

VitE and DEX in simultaneous form of administration significantly (P<0.05) down-regulated the tissue ALP level and attenuated the VCL-decreased GSH-px, SOD and TAC levels and remarkably (P<0.05) down-regulated the testicular malondialdehyde (MDA) and nitric oxide (NO) contents. The VCL-induced histopathological alterations significantly (P<0.05) improved in VitE and DEX-administrated animals. The VitE and DEX co-administration reduced the VCL-increased RNA damage and elevated the Leydig cells steroidogenic activity. The Hsp70-2 protein level completely (P<0.05) increased in VitE and DEX alone–and-simultaneous-administrated animals. Finally, the VitE and DEX could significantly (P<0.05) improve the VCL-decreased semen quality and improved the sperm DNA integrity and chromatin condensation.

**Conclusion:**

Our data suggest that Vit E by up-regulating the antioxidant status and DEX by reducing inflammation-dependent oxidative and nitrosative stresses could improve the VCL-reduced Hsp70-2 chaperone expression and ultimately protected the testicular endocrine activities and promoted the spermatogenesis process.

## INTRODUCTION

Varicocele (VCL) is characterized by a progressive disorder which is defined by tortuosity of the pampiniform plexus veins. It has been reported that the VCL is more commonly found in the left side, while it is able to exert bilateral impacts (1, 2). Although there are several reports about the VCL pathophysiology, the exact mechanism of impaired testicular function is not completely understood. Elevated oxidative stress (3), germinal cells apoptosis (4), nitrosative stress (5) and severe testicular inflammation (6) are reported for reasons of infertility in VCL patients. The relation between VCL-induced derangements associated with down-regulated antioxidant capacity of the testicular tissue and their correlations with venous stasis-induced hypoxia have been established in several reports (5, 6). Beside different stresses, the cytokines roles in steroidogenesis and over-expression of potent pro-inflammatory activators have been previously reported for VCL patients (7). Cytokines-induced polymorphonuclear cells infiltration during VCL-induced inflammation is known as the main cause of extracellular reactive oxygen (ROS) increase (6, 8). Therefore, the positive correlation between VCL-reduced antioxidant capacity and inflammation-induced ROS generation is not ignorable.

On the other hand, normal spermatogenesis largely depends on synthesis of several proteins in each type of special germ cell. Various families of heat shock proteins (Hsp) as Hsp70 and Hsp90 display crucial developmental roles in mammalian spermatogenesis (9, 10). It has been shown that the expression of Hsp70 families increases in response to elevated temperature and environmental stresses (11). Hassun Filho et al. showed the essential role of Hsp90 in spermatogenesis and reported that mutation of Hsp90 in varicocele men results in impaired spermatogenesis (12). In relation with this finding, the heat-dependent apoptosis is considered as one of important reasons for germ cells degeneration in VCL–induced testes (4), which illustrates the role of Hsp70 chaperones in regulating spermatogenesis. The Spermatocyte-specific HSP70-2 is expressed at high levels in pachytene spermatocytes during the meiotic phase of spermatogenesis (13, 14). The current chaperone interacts in several cell-specific pathways as assembling, folding, and refolding of proteins, DNA damages recovery and participating in specific RNA-binding proteins assembling (15, 16). The Hsp70-2 by promoting the suppression of pro-inflammatory immune responses interacts in various inflammatory reactions of reproductive (16) and digestive systems (17). However, the role of Hsp70-2 protein in VCL-induced inflammation and oxidative stress remains unknown.

In light of these findings and considering the regulatory role of Hsp70-2 in spermatogene-sis, the current study was undertaken to evaluate whether the co-administration of Dexamethasone (DEX), as anti-inflammatory agent, and vitamin E (VitE), as potent antioxidant, could prevent the VCL-induced damages in testicular tissue. For this purpose the testicular tissue total antioxidant capacity (TAC), tissue malondialdehyde (MDA) content, biochemical changes of superoxide dismutase (SOD), glutathione peroxidase (GSH-Px) in testicular tissue, the nitric oxide (NO), the histological features for germinal cells RNA damage, Leydig cells bioesteroid activities and sperm parameters were analyzed. Moreover, the protective effect of VitE and DEX on VCL-induced alterations in Hsp70-2 chaperone expression was evaluated as well. Moreover, this study was aimed to uncover their protective effect on sperm parameters.

## METHODS AND MATERIALS

### 

#### Animals

Thirty mature male Wistar rats, 10 weeks old and weighing between 180 to 200 g were used. The rats were obtained from the Animal House of Faculty of Veterinary Medicine, Urmia University (Iran) and were acclimatized in an environmentally controlled room (temperature, 20-22°C with 12h light/12h dark). Food and water were given ad libitum. In this study all experiments were in accordance with the Urmia University guidelines for research on laboratory animals.

#### Varicocele induction

In test groups left varicocele was induced as previously reported by Sofikitis and Miyagawa (18). In brief, following induction of anesthesia with ketamine 5% (Razak, Iran), 40 mg kg-1, i.p. and xylazine 2% (Trritau, Germany) 5 mg kg-1, i.p., a 2 cm median incision was performed and the diameter of left renal vein was reduced to 1 mm; the left renal vein ligation was performed medial to the junction of the adrenal and spermatic veins. Then the anastomotic branch between the left testicular vein and the left common iliac vein was ligated by 0-4 silk suture. The animals in control-sham group were anesthetized and only underwent to a simple laparotomy and no vein ligation was performed on these animals.

#### Experiment design and administration of compounds

Following one-week of acclimatization, the animals were assigned into five groups (n=6) including control-sham and test groups. A vehicle dose of 0.5 mL of saline (0.85% w/v) was administered to control-sham group by oral gavages and intraperitoneal route (n=3 for each route of administration). Following one week after VCL induction the test group was subdivided into 4 groups:

- Non-treated VCL-induced group.
- Vitamin E-received VCL-induced group; animals in this group received 150mg/kg^−1^b. wt. of VitE (Sigma Chemical Co. st Louis MO), by oral gavages (19).
- DEX-administrated VCL-induced group; the DEX was administrated at the dose of 0.125mg/kg^−1^/day, intraperitoneally (20).
- DEX and VitE-received VCL-induced group.

#### Testicular weight determination

Following 60 days, the animals were weighted for total body weight and the left testes were dissected out and weighted on a Mattler Bas-bal scale (Delta Range, Tokyo). The total testicular weight gains of left testes on total body weight gains were evaluated in all animals.

#### Histological analyses

The left testicular tissues were dissected out and washed with normal saline. Half of the tissues were fixed in Bouin's fixative for histological investigations and subsequently embedded in paraffin. Sections (5–6μm) were stained with Iron-Weigert (PajoheshAsia, Iran) for detection of germinal cell nuclei in the testis. The histological slides were analyzed under light microscope at two magnifications (×400 and ×1000). Analysis of the diameter of seminiferous tubules was done with calibrated lens. In brief, the average of the small and big diameter of each tubule was calculated using the formula magnification 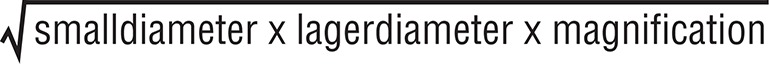. The tubular differentiation (TDI) and repopulation (RI) indices were evaluated in 20 sections of each sample. The results for percentage of tubules with positive TDI and RI were reported.

#### Fluorescent analyses for RNA damage

The RNA damage was assessed using the Acridine-orange NO dye (Sigma Aldrich, Germany) according to von Bertalanffy and Bickis method (21). In brief, the testes were washed out with ether alcohol and cut by cryostat (8μm). The prepared sections were fixed by different degrees of alcohol (ethanol) for 15 minutes. Then the sections were briefly rinsed in acetic acid, 1% aqueous, followed by washing in distilled water. The specimens then were stained with acridine-orange for 3 minutes and distained in phosphate buffer, followed by fluorescent colors differentiation in calcium chloride. The degenerated cells were characterized by loss of RNA and/or with faint red stained RNA. The normal cells manifested with bright red RNA at the apex of the nucleolus. In order to reduce the bias problems for staining density and for better evaluation of the RNA damage, 20 sections for each sample were investigated. The percentage of tubules with RNA damage was reported for all groups.

#### Assessment of Leydig cells steroid activity and serum level of testosterone

The Leydig cells biosteroid activity was investigated using commercially available kit for fluorescent assay of intracytoplasmic steroid droplets, Pajoheshasia (FLP, IUO100). In brief; the frozen section prepared slides were dehydrated and stained with hematoxylin. The slides were washed with running water for 3-5 minutes and stained with special fluorescent dye (FITC-conjugated1–anilinonaphthalene-8-sulphonate) for steroids and rinsed in distilled water. After rinsing, the slides were dehydrated in 95 and absolute isopropanol and mounted in Harleco fluorescent mountant.

#### Evaluating serum level of testosterone

In order to evaluate the serum level of testosterone the blood samples were collected directly from heart and serum samples were prepared by centrifugation (3000 g for 5 min), and the serum level of testosterone was assessed using a commercial kit for competitive chemiluminescent immunoassay (PishtazTeb, Iran).

#### Immunohistochemcal analyses for Hsp70-2

Tissue section slides were heated at 60°C for approximately 25 min in a hot air oven (Venti cell, MMM, Einrichtungen, Germany). The tissue sections were deparaffinized in xylene and rehydrated using an alcohol gradient. The antigen retrieval process was performed in 10 mM sodium citrate buffer. Immunohistochemical staining was conducted according to the manufacturer's protocol (Biocare, USA). Briefly, endogenous peroxidase was blocked in a peroxidase blocking solution (0.03% hydrogen peroxide containing sodium azide) for 5 min. Tissue sections were washed gently with washing buffer and subsequently incubated with Hsp70 (1:500) biotinylated primary antibodies for 15 min. The sections were rinsed gently with washing buffer and placed in a buffer bath. The slides were then placed in a humidified chamber with a sufficient amount of streptavidin - HRP (streptavidin conjugated to horseradish peroxidase in phosphate-buffered saline (PBS) containing an anti-microbial agent). The slides were incubated for 15 min. Subsequently, the tissue sections were rinsed gently in washing buffer and placed in a buffer bath. The AEC chromogen was added to the tissue sections and incubated for 5 min, followed by washing and counterstaining with hematoxylin for 5 sec. The sections were then dipped in weak ammonia (0.037 M/L) 10 times, rinsed with distilled water and cover slipped. Positive immunohistochemical staining was observed as brown stains under a light microscope.

#### Western blot analysis for Hsp70-2

Western blot analysis was performed with a mouse monoclonal antibody specific for hsp70 (C92F3A-5; Stress Gen, Victoria, BC, Canada). The testicular tissues were immediately frozen in liquid nitrogen, homogenized, and centrifuged. After determination of protein concentration with the bicinchoninic acid method, 100 mg of proteins in each sample was loaded onto a 7.5% SDS-PAGE system. The blots were transferred onto a PVDF membrane (Bio-Rad Laboratories, Richmond, CA) and incubated in Tris-buffered saline-Tween 20 (20.0 mM Tris-HCl, pH 7.5, 150.0 mM NaCl, 0.05% Tween 20) containing 2% skim milk to block nonspecific binding sites. The membrane was immune-reacted with a 1:5,000 dilution of MA3-009. Nitro blue tetrazolium and 5-bromo-4-chloro-3-indolyl phosphate were used as substrates for visualization of the reaction product. The degree of HSP70 expression was semi-quantitatively evaluated with computed densitometry (NIH Image; Macintosh; Apple Computers, Cupertino, CA).

#### Determination of tissue malondialdehyde (MDA), SOD, GSH-Px and TAC

For the biochemical evaluation of oxidant–antioxidant system, the testicular tissue was washed 3 times with 0.9% NaCl solution and 1.15% KCl was liquidified to amount of 9 ml for each tissue. The homogenate of the tissues was prepared with the teflon end on homogenizator (Elven-jempotter, Newton CT) and were centrifuged at 4000 rpm. The MDA content was measured using the thiobarbituric acid (TBA) reaction as described previously. The tissue SOD and GSH-pxactivities were evaluated using the measurement kits of RAN-SOD and RANSOL (Rondaxlab., Crumlin, BT 29, UK). We assessed the tissue TAC status based on the ferric reduction antioxidant power (FRAP) assay (22, 23). The protein level was estimated by the method of Lowry et al. (23).

#### Nitric oxide determination

As the direct measurement of nitric oxide (NO) in biological specimens is very difficult, the testicular tissue nitrite (NO^2^) was estimated as an index of NO production. The method for testis nitrite and nitrate levels was based on the Griess reaction (24). In brief; the samples were deproteinized with Somogyl reagent. Total nitrite (nitrite+nitrate) was measured after conversion of nitrate to nitrite by copporized cadmium granules by a spectrophotometer at 545 nm (Ultraspec Plus, Pharmacia LKB Biochrom Ltd, England). A standard curve was established with a set of serial dilutions (10^−8^ to 10^−3^mol/L) of sodium nitrite. Linear regression was done using the peak area from nitrite standard. The resulting equation then was used to calculate the unknown sample concentrations. Finally, the results were expressed as nanomole per gram of testis protein.

#### Analyzing epididymal sperm content and motility

The epididymis tissue was separated from the testicles under a 40-time magnification provided (by a stereo zoom microscope model TL2, Olympus Co., Tokyo, Japan). After separating the epididymis into three segments of caput, corpus and cauda, the epididymal cauda was trimmed and the content was added to 5 mL pre-warmed Hams F10 medium. After 20 min (incubating in CO_2_ incubator, LEEC, England) the epididymal tissue was separated from the released spermatozoa. The sperm count was performed according to standard hemocytometric method as described previously by Pant and Srivastava, 2003 (25).

In order to evaluate the sperm motility, the WHO [1999] standard method for manual examination of sperm motility was used (26). Briefly; the sperm samples were diluted (1:8) in Ham's F10 before examination. A 20μL of sperm sample was placed on sperm examination area and examined by 10× magnification loop. Only the motile sperm with forward progression counted within 10 boxes were recorded. Finally, motility was evaluated based on the following equation:


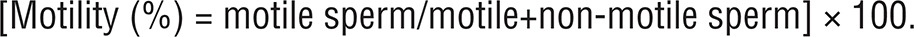


#### Evaluating sperm DNA damage and chromatin condensation

In order to evaluate the DNA double strand breaks the acridine orange staining kit (Poly Scientific R &D Crop, New York, USA) was used and analyzed by Epi-fluorescent microscope (Model GS7, Nikon co., Japan). The sperms with red and/or yellow fluorescent stained nucleus were considered as sperms with single strand DNA and the sperms with light green stained nucleus were marked as sperms with double strand DNA. The percentages of sperms with double strand DNA were reported.

The aniline-blue staining was performed in order to evaluate sperm chromatin condensation. Briefly; 5μL of the prepared spermatozoa were smeared, and allowed to dry in laboratory temperature. The smears were fixed in 3% buffered glutaraldehyde in 0.2 M phosphate buffer (pH 7.2) for 30 min. Slides were then stained with 5% aqueous aniline blue mixed with 4% acetic acid (pH 3.5) for 5 min and then 100 sperm cells per slide were evaluated and the percentage of unstained sperm heads was calculated. The unstained sperms were considered as sperms with condensed chromatin and the completely stained sperms were marked as uncondensed chromatin. Finally the percentages of sperms with condensed chromatin were reported.

#### Assessment of tissue alkaline phosphatase (ALP)

In order to evaluate the tissue levels of ALP the tissue was homogenized as previously described. Then, the tissue level of ALP was measured using the commercially available standard kit (ALP, 744, Man Inc. Tehran, Iran) according to manufacturer's introduction.

### Statistical analyses

The statistical analyses were performed on all numerical data by using two-way ANOVA and using SPSS software version 13.0. All values were expressed as the mean ± SD. P<0.05 was considered to be statistically significant.

## RESULTS

### 

#### General characteristics of treated and non-treated animals

**Figure 1:**
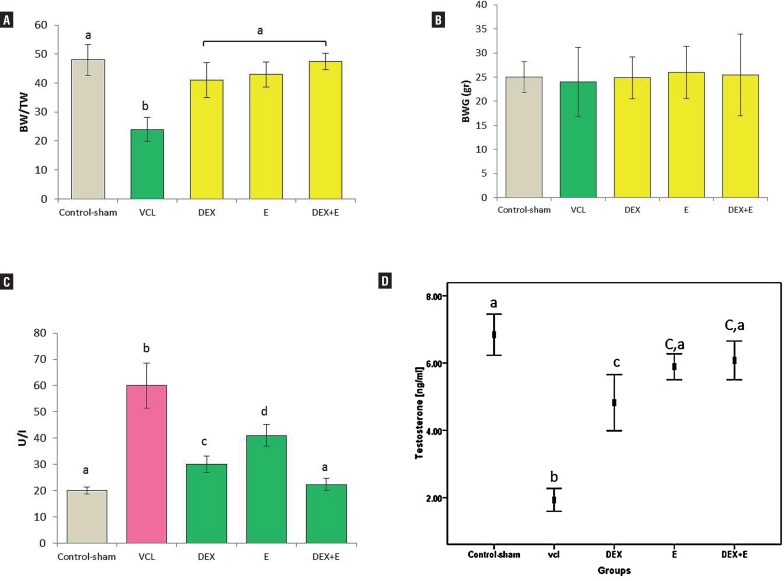
Effect of VitE (150mg/kg) and DEX (0.125mg/kg) on (A) VCL-reduced testicular weight (TW) to body weight (BW) ratio; (B) VCL-decreased body weight; (C) VCL-declined testicular level of ALP; and (D) VCL-decreased serum level of testosterone; the VitE and DEX co-administration managed to significantly (P<0.05) recover the VCL-induced derangements.

After 60 days, the total body weight of the animals did not changed in all treated and non-treated VCL-induced groups in comparison to control-sham group. The testicular to body weight ratio remarkably declined in VCL-induced animals versus the treated and control-sham groups (Figures 1-A,B). The serum level of testosterone significantly (P<0.05) declined in non-treated VCL-induced group compared to control-sham, while the DEX and VitE-alone and simultaneous administration significantly (P<0.05) improved the testosterone level. Accordingly, the animals in DEX+VitE-treated group showed the highest level of testosterone versus other test groups (Figure 1-C). Comparing the serum level of alkaline phosphatase (ALP) between all groups showed that the tissue level of ALP significantly (P<0.05) increased in non-treated VCL–induced groups in comparison to control-sham animals. Meanwhile, the DEX and VitE administration significantly declined the tissue level of ALP (Figure 1-D).

#### DEX and VitE simultaneous administration reversed the VCL-induced histological damages

**Figure 2:**
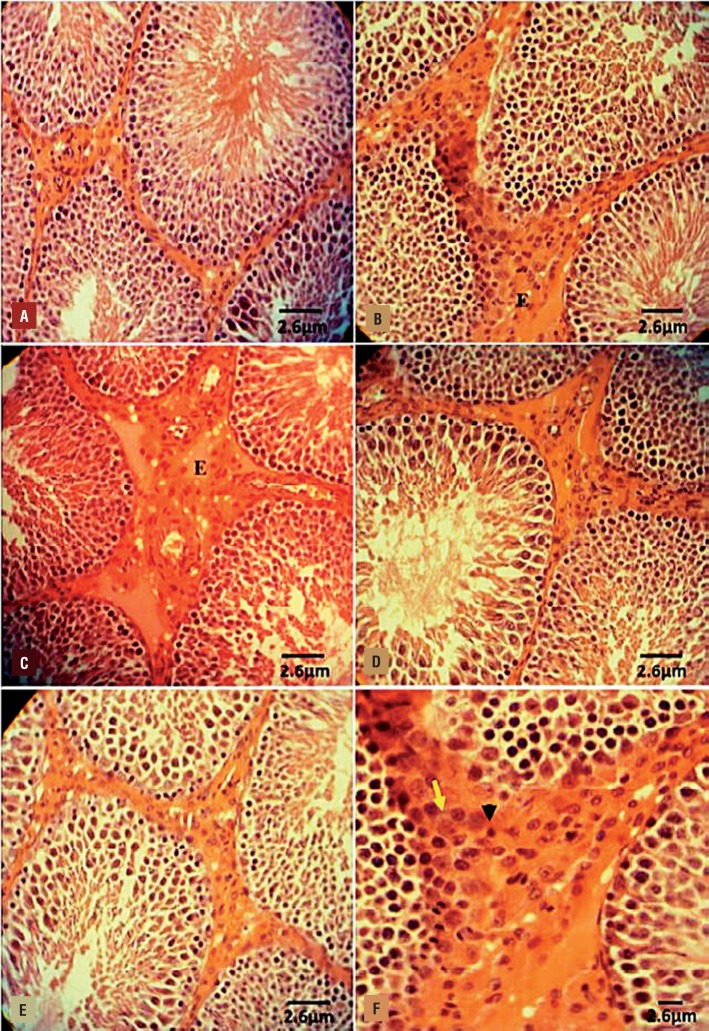
Photomicrograph of rat testes. (A) Control-sham group: normal germinal epithelium with no edematous connective tissue; (B) VCL-induced group: testicular tissue represented with severe edema (E) in connective tissue and remarkable immune cells infiltration in interstitial tissue; (C) VitE-administrated group: the protected seminiferous tubules with positive TDI and RI are observed, while the edematous interstitial tissue (E) is remained unchanged; (D) DEX received group: note decreased edema and down regulated immune cells infiltration. The seminiferous tubules are presented with normal TDI and RI; (E) VitE+DEX-administrated group: the edema and immune cells infiltration significantly reduced versus figures D and E, and the tubules are presented close to normal; and (F) Higher magnification from interstitial tissue of non-treated VCL-induced testis: note mononuclear immune cell (head arrow) infiltration in edematous tissue and hypertrophied leydig cells (arrow) distribution. Iron-Weigert staining technique (×400).

The histological analyses revealed a normal structure in the testis of the control-sham animals. While the non-treated VCL-induced animals manifested with arrested spermatogenesis, negative TDI, RI and severe edema in the interstitial connective tissue. In contrast, the DEX and VitE–administrated animals showed improved spermatogenesis, reduced percentage of tubules with negative TDI, RI, remarkably reduced edema and most importantly decreased mononuclear immune cells infiltration (Figures 2 A-F). The data for morphometric analyses are presented in Table-1.

**Table 1:** Summary of histological analyses; all data are given as Mean±SD (n=6).

	T.D (µm)	G.E.H (µm)	G.E.D (%)	RI (%)	TDI (%)
Control-sham	387.43±18.80^a^	201.10±13.91^b^	280.41±16.00^c^	276.15±12.28^c^	331.11±17.93^d^
VCL	271.44±20.11^a^	134.22±15.51^b^	186.71±23.77^c,e^	176.51±16.41^c^	221.44±14.00^d,e^
VCL+E	3.44±1.33^a^	57.32±8.81^b^	21.15±5.90^c,e^	25.10±2.55^c^	12.41±3.60^d,e^
VCL+DEX	7.89±1.11^a^	56.66±6.21^b^	38.91±1.86^c^	30.43±2.90^c^	19.11±3.23^d^
VCL+E+DEX	9.36±1.23^a^	62.41±7.84^b^	35.52±4.11^c^	37.12±5.71^c^	22.21±4.55^d^

Average of histomorphometric analyses for mean percentage of tubular depletion (T.D), germinal epithelium height (G.E.H) mean percentage of tubules with germinal epithelium dissociation (G.E.D), mean percentage of seminiferous tubules with negative tubular differentiation (TDI) and repopulation indexes (RI).

**Note:** E: vitamin E (150mg/kg), DEX: dexamethasone (0.125mg/kg), VCL: varicocele.

^a,b,c,d,e^: indicate significant differences (P<0.05) between marked data in same row.

#### DEX and VitE declined the VCL-increased RNA damage

**Figure 3:**
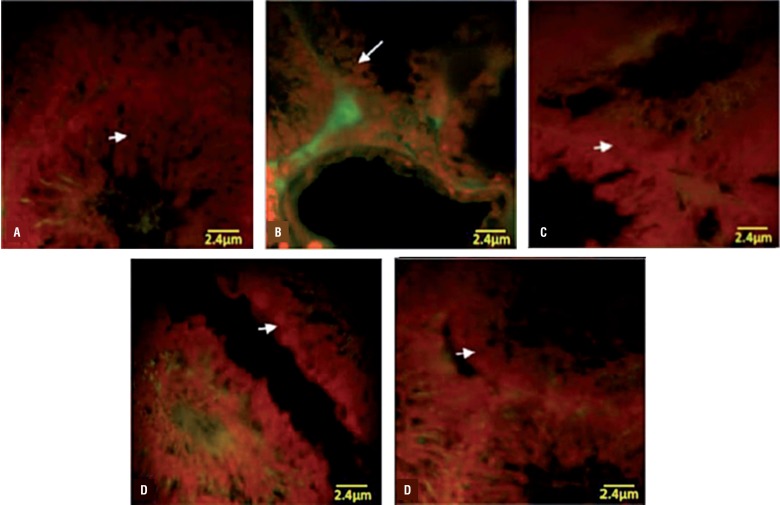
Fluorescent photomicrograph of testes. (A) control-sham group: the germinal cells in different layers are presented with remarkably higher normal mRNA levels (arrow); (B) VCL induced group: severe RNA damage has been marked with faint yellowish red appearance (arrow); (C) VitE-treated; (D) testosterone-received; (E) VitE+testosterone-administrated groups; simultaneous administration of VitE and testosterone remarkably inhibited mRNA damage (arrows). Fluorescent staining for mRNA (×400).

**Figure 4:**
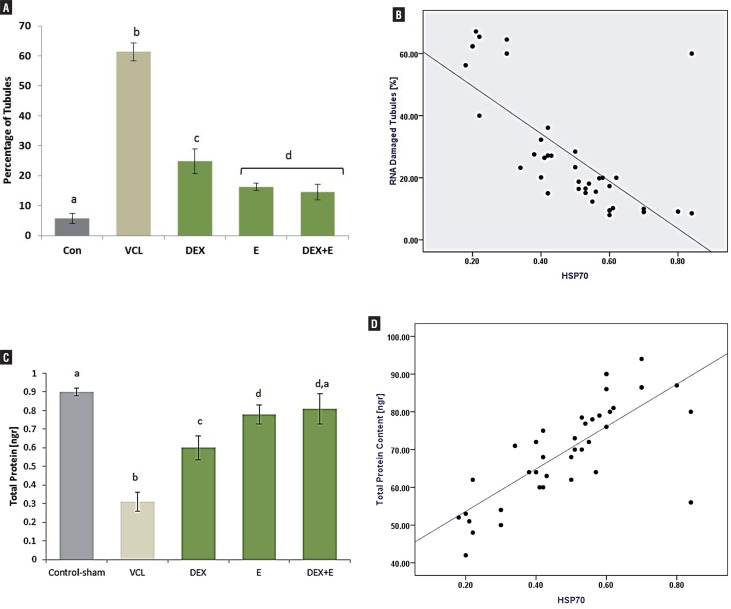
Effect of VitE (150mg/kg) and DEX (0.125mg/kg) on (A) VCL-increased percentage of tubules with damaged RNA, Vit E and DEX significantly (P<0.05) reduced the VCL-increased RNA damage; (B) Positive correlation between intensity of Hsp70-2 and percentage of tubules with RNA damage in germinal cells (r2 =0.876, P<0.05); (C) Effect of VitE and DEX on testicular total protein level, simultaneous administration of DEX and VitE significantly (P<0.05) elevated testicular protein level; and (D) Positive correlation between intensity of Hsp70-2 with testicular total protein level (r2 = 0.941, P<0.05).

The fluorescent analyze was performed to examine the RNA damage in germinal cells. The pathological effect of VCL after 60 days resulted in severe RNA damage in all germinal epithelium layers, which was characterized with faint red fluorescent appearance (Figures 3 A-E). The percentage of tubules with RNA damage in non-treated VCL groups was significantly (P<0.05) higher than that of control-sham. Unlike the VCL-induced testes, the DEX and VitE alone-and- simultaneously treated animals exhibited lower percentage of tubules with degenerated RNA (Figure 4-A).

#### DEX and VitE co-administration inhibited VCL–induced impact on Leydig cells

**Figure 5:**
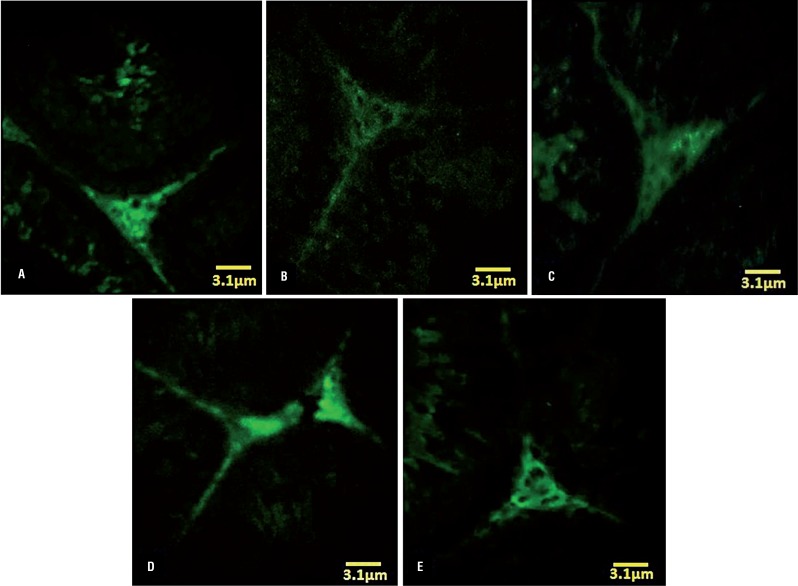
Fluorescent photomicrograph for leydig cells estroidogenesis. (A) Control-sham group: the leydig cells are marked with intensive intacytoplasmic steroid (arrow), (B) Non-treated VCL group: the leydig cells are presented with faint steroidogenic activity; (C) VitE-received group: the VitE improved the steroidogenic activity of leydig cells versus non-treated VCL-induced testis (arrows); (D) DEX-treated; (E) VityE+DEX-administrated group: note the improved steroidogenesis in these groups, which leydig cells are presented close to normal in control-sham testis. Fluorescent staining for leydig cells steroidogenesis (×400); and (F) Effect of Vit E (150 mg/kg) and DEX (0.125mg/kg) on VCL-decreased percentage of leydig cells with steroidogenic activity, the VitE and DEX co-administration remarkably (P<0.05) improved leydig cells endocrine activity.

The Leydig cells distribution (per one mm2 of the connective tissue) significantly (P<0.05) de creased in VCL group (18.75±3.41) compared to control-sham (35.62±6.42). Meanwhile, the DEX alone (26.41±4.16), VitE alone (29.31±4.32) and DEX+VitE-simultaneous-treated (31.22±1.28) groups revealed with remarkably (P<0.05) higher Leydig cells distribution. On the other hand, DEX+VitE-treated group showed significantly (P<0.05) higher percentage of Leydig cells with steroidogenic activities compared to non-treated VCL-induced animals (Figures 5 A-F). Moreover, the percentage of hypertrophied Leydig cells significantly (P<0.05) elevated in non-treated VCL animals versus the treated groups. In contrast, the animals in DEX+VitE group exhibited the lowest percentage of hypertrophied Leydig cells (Table-2).

**Table 2:** Effect of Vit E and DEX on VCL-induced derangements on Leydig cells; all data are given as Mean±SD (n=6).

	Leydig cells (NO/one mm^2^)	Hypertrophied Leydig cells (%)
Control-sham	35.62±4.42^a^	5.23±1.06^a^
VCL	18.75±3.41^b^	32.44±4.61^b^
VCL+E	29.31±4.32^a^	10.10±2.03^c,e^
VCL+DEX	26.41±4.16^a^	13.11±1.65c
VCL+E+DEX	31.22±1.28^a^	7.63±1.07^d,e^

**Note:** E: vitamin E (150mg/kg), DEX: dexamethasone (0.125mg/kg), VCL: varicocele.

^a,b,c,d,e^: indicate significant differences (P<0.05) between marked data in same column.

#### DEX and VitE simultaneous administration regulated the VCL-reduced Hsp70-2 expression in testis

**Figure 6:**
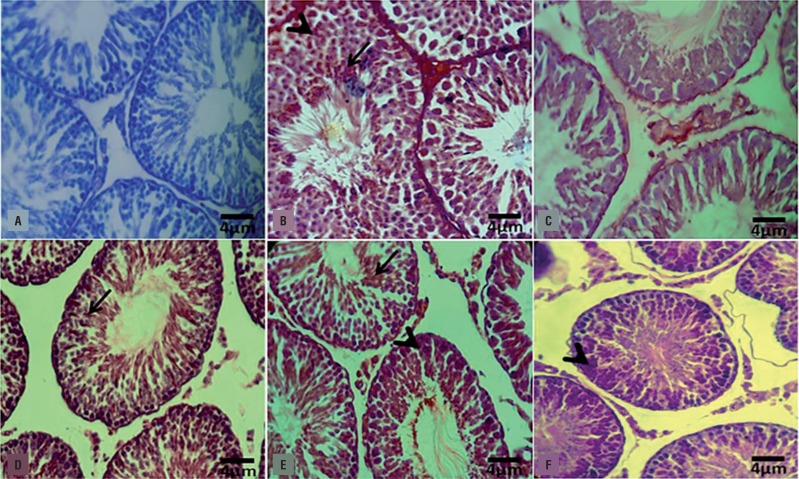
Immunohistochemical analyses for Hsp70-2. (A) negative control; (B) control-sham: intensive Hsp70-2 expression is presented in pachytene spermatocytes (head arrow) and in the cell in spermiogenesis process (arrow); (C) VCL-induced group: the germinal cells are exhibited with remarkable reduction in Hsp70-2 expression; (D) VitE-treated group; (E) DEX-received group; and (F) VitE+DEX-administrated group: the Hsp70-2 expression was back to control condition, which the spermatocyte (head arrow) and spermatids (arrow) are presented with up regulated Hsp70-2 expression. Immunohistochemical staining for Hsp70-2 (×600).

**Figure 7:**
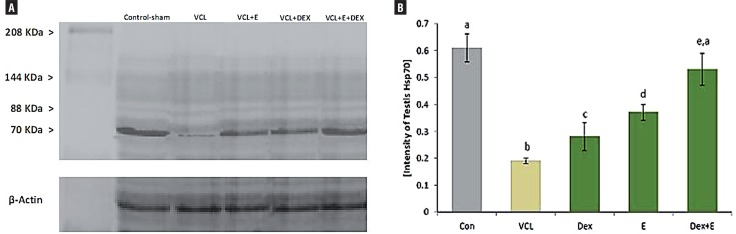
Western blot analyze for Hsp70-2 protein expression in testicular extract. The VitE (150 mg/kg) and DEX (0.125 mg/kg) alone and simultaneous administration enhanced the Hsp70-2 protein expression as there is no significant difference (P>0.05) between VitE+DEX-treated animals with control-sham group.

Immunohistochemical analysis was performed to evaluate the Hsp70-2 expression in different layers of germinal epithelium. Observation demonstrated that the Hsp70-2 chaperone expression significantly (P<0.05) decreased in non-treated VCL-induced testes. In contrast, the DEX and VitE regulated the HSP70-2 expression after 60 days (Figures 6 A-F). The western blot analyses confirmed the immunohistochemical examination. Accordingly, the animals in DEX+VitE–treated group exhibited significantly (P<0.05) higher HSP70-2 protein intensity compared to non-treated VCL, DEX and VitE-alone administrated groups. More statistical analyses revealed that there was a positive correlation between decreased HSp70-2 expression with decreased total protein content and the percentage of tubules with damaged RNA (Figures 7 A,B and Figures 4-B,C,D).

#### DEX and VitE elevated VCL-reduced TAC, GSH-px, SOD and reduced the VCL-increased MDA level

The testicular levels of TAC, GSH-px and SOD were measured in different groups. The non-treated VCL group presented significant (P<0.05) decrease in testicular TAC, GSH-px and SOD levels in comparison to control-sham animals. While the DEX and VitE-administrated groups remarkably up-regulated the GSH-px and SOD contents associated with testicular TAC level. Moreover, the DEX+VitE-administration significantly (P<0.05) declined the VCL-elevated MDA content (Table-3).

**Table 3:** Effect of Vit E and DEX on VCL-induced changes in antioxidant status and MDA level; all data are given as Mean±SD (n=6).

	GSH-px	SOD	TAC	MDA
	(U/mg protein)	(U/mg protein)	(mmoL/mg protein)	(nmoL/mg protein)
Control-sham	35.00±4.60^a^	700.66±15.04^a^	0.51±0.013^a^	1.41±0.05^a^
VCL	10.46±2.10^b^	174.53±22.50^b^	0.11±0.015^b^	9.21±0.26^b^
VCL+E	28.22±1.35^c,e^	608.45±35.61^cf^	0.42±0.012^c^	4.70±0.34^c^
VCL+DEX	24.22±4.06^c^	546.36±40.02^d^	0.37±0.010^d^	4.83±0.40^c^
VCL+E+DEX	31.26±2.28^a,d,e^	651.11±16.51^e,f^	0.49±0.011^e^	2.36±0.12^d^

Note: **E:** vitamin E (150mg/kg), **DEX**: dexamethasone (0.125mg/kg), **GSH-px**: glutathione peroxidase, **SOD**: superoxide dismutase, **TAC**: total antioxidant capacity, **MDA**: malondialdehyde, **VCL**: varicocele.

^a,b,c,d,e,f^: indicate significant differences (P<0.05) between marked data in same column.

#### Effects of DEX and VitE on VCL-induced nitrosative stress

**Figure 8:**
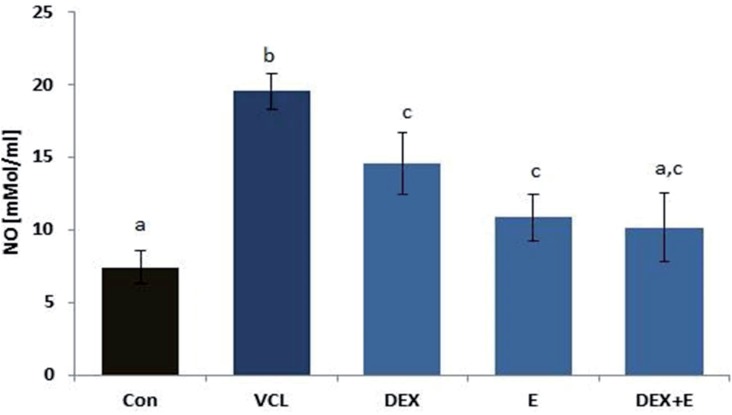
Effect of VitE (150mg/kg) and DEX (0.125 mg/kg) on VCL-elevated NO level, the co-administration of VitE and DEX significantly (P<0.05) reduced testicular NO level versus non-treated animals.

Biochemical observations demonstrated that VCL resulted in severe elevating of NO in testicular tissue versus control-sham group. Meanwhile, the treated animals exhibited significantly (P<0.05) reduced levels of NO in testicular tissue. Accordingly, the animals in DEX+Vit E–simultaneous-administrated group showed the lowest NO content compared to DEX and VitE alone-administrated animals (Figure-8).

#### DEX and VitE protected the VCL-induced damages in sperm parameters

**Figure 9:**
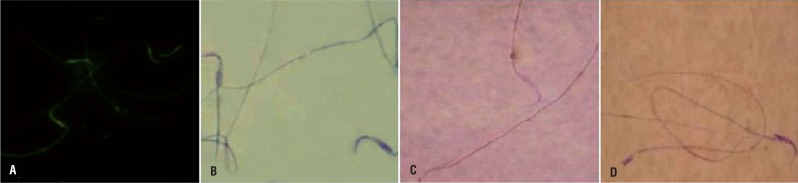
Representative profile of sperms in different staining techniques. (A) Acridine-orange staining for sperm DNA integrity: light green stained sperm is representing sperm cell with double strand DNA and the sperms with damaged DNA are presented with bright red stained nucleus; (B) Aniline-blue staining for chromatin condensation: the sperm with condensed chromatin is presented with light stained nucleus and the sperm with dark stained nucleus is representing de-condensed chromatin; (C); and (D) Eosin-nigrosin staining for sperm viability: live sperms are presented with colorless cytoplasm in figure (C) and the dead sperms are represented darkly stained cytoplasm in figure (D).

The non-treated VCL-induced animals showed a significant (P<0.05) decrease in sperm count compared to control-sham and treated groups. Fluorescent staining for DNA damage indicated that VCL-induced sperm damage shown in Figure 9-A was significantly ameliorated by DEX and VitE administration (P<0.05). The aniline-blue staining for sperm chromatin condensation revealed that the animals in DEX+VitE-administrated group (76.50±3.41) exhibited significantly (P<0.05) higher percentage of sperms with nuclear maturity in comparison to non-treated VCL-induced (52.75±4.99), DEX alone-administrated (63.00±2.94) and VitE alone-received (66.50±3.06) groups. No changes in sperm count and chromatin condensation were observed in control-sham group (Figure 9-B). The sperm viability remarkably (P<0.05) decreased in non-treated VCL-induced animals, while the co-administration of DEX and VitE elevated the sperm viability after 60 days (Figures 9 C,D). Similar to other sperm parameters, the treated animals illustrated with significant (P<0.05) elevation in sperm motility versus the non-treated ones. The data for sperm parameters are presented in Table-4.

**Table 4:** Regulating effect of VitE and DEX on VCL-changed sperm parameters; all data are given as Mean±SD (n=6).

	Control-sham	VCL	VCL+E	VCL+DEX	VCL+E+DEX
Count (×10^6^)	65.33±3.09^a^	14.35±3.17^b^	47.75±3.18^c^	40.62±4.10^c^	58.73±6.20^d^
Motility (%)	92.57±3.00^a^	19.75±2.74^b^	77.76±3.23^c^	68.41±4.09^d^	86.42±3.10^e^
DNA Damage (%)	6.41±1.42^a^	71.00±8.41^b^	35.00±4.60^c^	40.14±4.03^c^	26.07±2.03^d^
Chromatin Condensation (%)	89.42±6.12^a^	18.06±2.10^b^	58.41±6.42^c,e^	51.11±5.76^c^	64.31±4.08^d,e^

**Note**: E: vitamin E (150mg/kg), DEX: dexamethasone (0.125mg/kg), VCL: varicocele.

^a,b,c,d,e^: indicate significant differences (P<0.05) between marked data in same row.

## DISCUSSION

This study reports for the first time that the simultaneous administration of VitE and DEX is able to protect the testicular tissue and the sperm parameters against the VCL-induced damages via up-regulating the Hsp70-2 expression. The ameliorative effects of these two compounds were shown in the VCL-induced nitrosative and oxidative stresses, testicular endocrine activities, histopathological changes and RNA damage. There are different clinical reports in which varicocelectomy have been identified as an appropriate method in order to treat infertility (27, 28). On the other hand, some studies showed that administrating different antioxidant compounds can inhibit the VCL-induced damages. Accordingly, earlier study conducted by Lenzi and co-workers showed that Gluthatione (GSH) exerted positive and statistically significant effects on sperm parameters, as it improved sperm count and motility in 29 VCL patients (29). Another study showed that administrating 2 g day^−1^ of carnitine in 219 men with varicocele improved sperm morphology, motility and concentration after 4 months (30). Lastly, we showed that 50 mg/kg day^−1^ of sylimarin significantly decreased VCL-induced DNA damage and up-regulated the sperm motility and concentration in rats (31). On another study we showed that simultaneous administration of vitamin E and testosterone positively and remarkably reduced VCL–induced damages at testicular tissue and sperm level (30). Therefore, these findings enhance the hopes about reducing the VCL-induced derangements by medical interventions.

We lastly showed that the silymarin was able to elevate the VCL-reduced testicular weight (31). Current study showed that the simultaneous administration of the VitE and DEX was able to preserve the testicular to total body weight ratio more significant than that of silymarin as a unique antioxidant compound. This finding suggests that co-administration of an anti-inflammatory compound with an antioxidant agent leads to better result, which may be attributed to lower testicular degeneration.

Previous studies (31, 32) illustrated the VCL–induced inflammation in testicular tissue (6, 8) of animal models. Current study showed that administration of DEX significantly reduced the VCL–increased ALP and remarkably down-regulated the severe immune cells infiltration. Considering that the monocytes, neutrophils and macrophages are known as the main sources of cytokines in testicular tissue (33–35), we can conclude that the administration of DEX resulted in remarkable reduction in cytokines level by reducing the immune cells infiltration. On the other hand, the systemic and local inflammations due to different causes result in remarkable reduction in the testicular steroidogenesis and inhibit the spermatogenesis infertilities (32). The relevance between inflammation-induced oxidative stresses and the enhanced damages from evacuation of testosterone in Leydig cells is mutual. Indeed, the infammation results in oxidative stress, which leads to remarkable damages at Leydig cells level. Consequently, the damaged Leydig cells loss their potential to synthesis enough testosterone for normal spermatogenesis, which in turn results in producing more ROS in testicular tissue (5, 31, 32). Our biochemical analyses showed that the VitE–administrated animals exhibited up-regulated antioxidant status with decreased MDA content. Moreover, the Leydig cells were manifested with increased steroidogenic activity, which proofed with elevated testosterone level. Thus, we can conclude that the VitE ameliorated the testicular endocrine activities via enhancing the antioxidant capacity, which is partly different with the DEX promoted mechanism.

Comparing the isolated and simultaneous form of administration revealed that the simultaneous administration of DEX and VitE exerted better results for enzymatic and non-enzymatic antioxidant defense lines and showed significantly higher levels of testosterone. This situation suggests the necessity of the VitE with DEX co-administration in order to inhibit the VCL induced damages.

**Figure 10:**
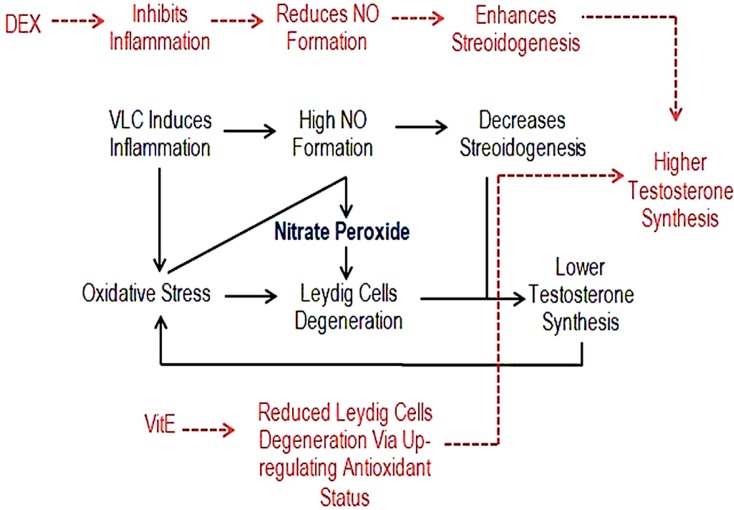
The VCL decreases testicular endocrine function by promoting nitrosative and oxidative stresses, which lead to severe damages at spermatogenesis and enhance the DNA and RNA damage. While, VitE reduces Leydig cells degeneration via up-regulating antioxidant status and DEX inhibits the NO generation by reducing the VCL-induced inflammation. Therefore, combination of these two compounds up-regulates the testicular endocrine activity.

In fact, the VCL-induced free radicals promote the NO peroxidation and result in nitrate peroxide formation, which will enhance the damages in testicular tissue (33, 35). The generated Oxidative and NO-dependent stresses result in severe damages at DNA, RNA and proteins levels (31, 36, 37). Therefore, preventing the NO generation by DEX and inhibiting its peroxidation by down-regulating the VCL-induced oxidative stress via VitE are able to decrease the damages in testicular tissue. This hypothesis was proofed with our findings. Accordingly, our biochemical analyzes showed that, the animals in treated groups (especially in Vit E+DEX-received group) presented decreased NO content. These findings were supported by histopathological observations such as enhanced steroidogenic activities of Leydig cells as well as up-regulated TDI and RI indices in DEX and VitE co-administrated group (Figure-10).

To maintain the homeostasis under different stresses, the germinal cells produce high levels of chaperones such as Hsp70-2, which is involved in assisting other proteins in their assembling, folding and refolding processes (10, 38). Considering the positive correlation between synthesis of GSH-px and SOD with expression of Hsp70-2 (10), it would be more logic to conclude that the remarkable elevation of GSH-px and SOD enzymes level in VitE and DEX-received animals might be attributed to increased expression of Hsp70-2 in testicular tissue. Thus, we can come close to this fact that the VitE and DEX ameliorated the VCL–induced stresses by two mechanisms; a- partly by reducing ROS producing sources as inflammatory and damaged cells and b- by up-regulating the VCL decreased synthesis of antioxidant enzymes via enhancing the Hsp70-2 protein expression. Indeed, the enzymes as proteins need the Hsp70-2 chaperones to refold and assemble following the free radicals-induced damages.

For the first time we showed severe RNA damage in germinal epithelium of the tubules, which presented normal in simple and routine staining. The fluorescent analyses revealed that more than 30% of the tubules in non-treated VCL–induced animals exhibited remarkable damage at RNA level, which decreased into lower than 15% after simultaneous administration of DEX and VitE. The chaperones in Hsp70 family participate in signal transduction pathway that regulates the RNA expression, transcription factors and proteins synthesis (38, 40). It has been showed that the RNA remains stable for up to 7 days in haploid germinal cells. The special RNA-binding proteins are involved in maintaining the stability of mRNA through recognition of a specific nucleotide sequence (14, 41). The assembling and folding of these proteins are largely dependent on Hsp70-2 expression in haploid cells (14). Thus, we can conclude that the VitE and DEX co- administration reduced the RNA damage via up-regulating the Hsp70-2 expression. In this regard, the enhanced Hsp70-2 synthesis maintained the RNA-binding proteins and promoted the synthesis of essential proteins for RNA stability.

The DEX+VitE-administrated animals revealed with significantly elevated percentage of sperms with chromatin condensation and normal DNA content. Presenting the chromosome core proteins such as transition proteins 1 and 2, and protamines 1 and 2 in post meiotic phases is needed to sperm nuclear maturity. The synthesis of these proteins largely depends on Hsp-70 proteins involvement (38). Therefore, any lack in Hsp70-2 expression can significantly affect the sperm chromatin condensation process, which in turn results in remarkable DNA damage in sperm cells (36, 37). Due to this fact, we can suggest that the DEX and VitE co-administration could reduce the sperm DNA damage not only by increasing the antioxidant status but also the compounds improved the chromatin condensation, as well.

According to previous reports the increased ROS generation results in remarkable reduction in sperm motility. Accordingly, generated ROS leads to intensive decrease in axonemal proteins phosphorylation and inhibits the activation of enzymes such as glucose 6-phosphate dehydrogenase (42, 44). We showed that simultaneous administration of VitE and DEX significantly enhanced the sperm motility versus non-treated VCL-induced animals. Therefore, it could be formulated the hypothesis that decreased oxidative stress associated with elevated antioxidant status following administration of Vit E and DEX inhibited the VCL-induced impacts on sperm motility.

## CONCLUSIONS

The data from the present study declared that the VitE and DEX simultaneous administration could clearly inhibit the VCL-induced damages in testicular tissue and improve the sperm parameters. Comparing the co-and-isolated administration of VitE and DEX clarified that the simultaneous form of administration exhibited better results in Hsp70-2 proteins expression and consequently down-regulated the VCL-induced RNA damage in germinal epithelium and significantly reduced the sperm DNA damages by promoting the chromatin condensation processes. Moreover, mechanisms of action for VitE and DEX may be attributed to their ameliorative impact on antioxidant status and down-regulating the VCL-induced inflammation. Whereof, the treated animals revealed with up-regulated synthesis of enzymes involved in antioxidant defense and with elevated number of Leydig cells with steroidogenic activity.

## References

[1] Naughton CK, Nangia AK, Agarwal A (2001). Pathophysiology of varicoceles in male infertility. Hum Reprod Update.

[2] Razi M, Sadrkhanloo RA, Malekinejad H, Sarafzadeh-Rezaei F (2011). Varicocele Time-dependently Affects DNA Integrity of Sperm Cells: Evidence for Lower In vitro Fertilization Rate in Varicocele-positive Rats. Int J Fertil Steril.

[3] Hendin BN, Kolettis PN, Sharma RK, Thomas AJ, Agarwal A (1999). Varicocele is associated with elevated spermatozoal reactive oxygen species production and diminished seminal plasma antioxidant capacity. J Urol.

[4] Smith R, Kaune H, Parodi D, Madariaga M, Rios R, Morales I (2006). Increased sperm DNA damage in patients with varicocele: relationship with seminal oxidative stress. Hum Reprod.

[5] Romeo C, Ientile R, Impellizzeri P, Turiaco N, Teletta M, Antonuccio P (2003). Preliminary report on nitric oxide-mediated oxidative damage in adolescent varicocele. Hum Reprod.

[6] Evers JH, Collins J, Clarke J (2008). Surgery or embolisation for varicoceles in subfertile men. Cochrane Database Syst Rev.

[7] Nallella KP, Allamaneni SS, Pasqualotto FF, Sharma RK, Thomas AJ, Agarwal A (2004). Relationship of interleukin-6 with semen characteristics and oxidative stress in patients with varicocele. Urology.

[8] French DB, Desai NR, Agarwal A (2008). Varicocele repair: does it still have a role in infertility treatment?. Curr Opin Obstet Gynecol.

[9] Zakeri ZF, Wolgemuth DJ, Hunt CR (1988). Identification and sequence analysis of a new member of the mouse HSP70 gene family and characterization of its unique cellular and developmental pattern of expression in the male germ line. Mol Cell Biol.

[10] Kaur P, Bansal MP (2003). Effect of oxidative stress on the spermatogenic process and hsp70 expressions in mice testes. Indian J Biochem Biophys.

[11] Hassun PA, Cedenho AP, Lima SB, Ortiz V, Srougi M (2005). Single nucleotide polymorphisms of the heat shock protein 90 gene in varicocele-associated infertility. Int Braz J Urol.

[12] Nakai A, Morimoto RI (1993). Characterization of a novel chicken heat shock transcription factor, heat shock factor 3, suggests a new regulatory pathway. Mol Cell Biol.

[13] Sarge KD, Park-Sarge OK, Kirby JD, Mayo KE, Morimoto RI (1994). Expression of heat shock factor 2 in mouse testis: potential role as a regulator of heat-shock protein gene expression during spermatogenesis. Biol Reprod.

[14] Hecht WB, Hansson V., Levy F.O., Tasken K. (1996). Post transcriptional regulation of post meiotic gene expression. Signal Transduction in Testicular Cells.

[15] Zhu D, Dix DJ, Eddy EM (1997). HSP70-2 is required for CDC2 kinase activity in meiosis I of mouse spermatocytes. Development.

[16] Neuer A, Spandorfer SD, Giraldo P, Dieterle S, Rosenwaks Z, Witkin SS (2000). The role of heat shock proteins in reproduction. Hum Reprod Update.

[17] Asano T, Tanaka K, Yamakawa N, Adachi H, Sobue G, Goto H, Takeuchi K, Mizushima T (2009). HSP70 confers protection against indomethacin-induced lesions of the small intestine. J Pharmacol Exp Ther.

[18] Sofikitis N, Miyagawa I (1992). Experimental models for the study of varicocele: A selected review. Jpn J Fertil Steril.

[19] Yousef MI, Abdallah GA, Kamel KI (2003). Effect of ascorbic acid and Vitamin E supplementation on semen quality and biochemical parameters of male rabbits. Anim Reprod Sci.

[20] Eken H, Ozturk H, Ozturk H, Buyukbayram H (2006). Dose-related effects of dexamethasone on liver damage due to bile duct ligation in rats. World J Gastroenterol.

[21] Bickis I, Von Bertalanffy L (1956). Identification of cytoplasmic basophilia (ribonucleic acid) by fluorescence microscopy. J Histochem Cytochem.

[22] Niehaus WG, Samuelsson B (1968). Formation of malonaldehyde from phospholipid arachidonate during microsomal lipid peroxidation. Eur J Biochem.

[23] Lowry OH, Rosebrough NJ, Farr AL (1995). Protein measurement with the Folin phenol reagent. J Biol Chem.

[24] Cortas NK, Wakid NW (1990). Determination of inorganic nitrate in serum and urine by a kinetic cadmium-reduction method. Clin Chem.

[25] Pant N, Srivastava SP (2003). Testicular and spermatotoxic effects of quinalphos in rats. J Appl Toxicol.

[26] WHO (1999). WHO laboratory manual for the examination of human semen and sperm cervical mucus interaction.

[27] Mostafa T, Anis TH, El-Nashar A, Imam H, Othman IA (2001). Varicocelectomy reduces reactive oxygen species levels and increases antioxidant activity of seminal plasma from infertile men with varicocele. Int J Androl.

[28] Hurtado de Catalfo GE, Ranieri-Casilla A, Marra FA, de Alaniz MJ, Marra CA (2007). Oxidative stress biomarkers and hormonal profile in human patients undergoing varicocelectomy. Int J Androl.

[29] Lenzi A, Culasso F, Gandini L, Lombardo F, Dondero F (1993). Placebo-controlled, double-blind, cross-over trial of glutathione therapy in male infertility. Hum Reprod.

[30] Khosravanian N, Razi M, Farokhi F, Khosravanian H (2014). Testosterone and vitamin E administration up-regulated varicocele-reduced Hsp70-2 protein expression and ameliorated biochemical alterations. J Assist Reprod Genet.

[31] Moshtaghion SM, Malekinejad H, Razi M, Shafie-Irannejad V (2013). Silymarin protects from varicocele-induced damages in testis and improves sperm quality: evidence for E2f1 involvement. Syst Biol Reprod Med.

[32] Razi M, Sadrkhanloo RA, Malekinejad H, Sarrafzadeh-Rezaei F (2012). Testicular biohistochemical alterations following experimental varicocele in rats. Iran J Reprod Med.

[33] Cheville NF (1999). Introduction to Veterinary Pathology.

[34] Baggiolini M, Dewald B, Moser B (1999). Chemokines. In Inflammtion Basic principles and clinical correlates.

[35] Cunha FQ, Ferreira SH (2003). Peripheral hyperalgesic cytokines. Adv Exp Med Biol.

[36] Razi M, Malekinejad H, Sadrkhanlou RA, Sarrafzadeh-Rezaie F (2011). Histological Impact of Long Term Varicocele-Induction on Right and Left Testes in Rat (Evidence for the Reduction of Sperm Quality and Mating Abilities). Vet research Forum.

[37] Malekinejad H, Janbaz-Acyabar H, Razi M, Varasteh S (2012). Preventive and protective effects of silymarin on doxorubicin-induced testicular damages correlate with changes in c-myc gene expression. Phytomedicine.

[38] Eddy EM (1999). Role of heat shock protein HSP70-2 in spermatogenesis. Rev Reprod.

[39] Eddy EM (1998). Regulation of gene expression during spermatogenesis. Semin Cell Dev Biol.

[40] Kwon YK, Hecht NB (1991). Cytoplasmic protein binding to highly conserved sequences in the 3’ untranslated region of mouse protamine 2 mRNA, a translationally regulated transcript of male germ cells. Proc Natl Acad Sci U S A.

[41] Kessopoulou E, Tomlinson MJ, Barratt CL, Bolton AE, Cooke ID (1992). Origin of reactive oxygen species in human semen: spermatozoa or leucocytes?. J ReprodFertil.

[42] Twigg J, Irvine DS, Houston P, Fulton N, Michael L, Aitken RJ (1998). Iatrogenic DNA damage induced in human spermatozoa during sperm preparation: protective significance of seminal plasma. Mol Hum Reprod.

